# An evaluation of anesthetic fade in motor evoked potential monitoring in spinal deformity surgeries

**DOI:** 10.1186/s13018-018-0934-7

**Published:** 2018-09-05

**Authors:** Ryo Ugawa, Tomoyuki Takigawa, Hiroko Shimomiya, Takuma Ohnishi, Yuri Kurokawa, Yoshiaki Oda, Yasuyuki Shiozaki, Haruo Misawa, Masato Tanaka, Toshifumi Ozaki

**Affiliations:** 1 Department of Orthopedic Surgery, Okayama University Hospital, 2-5-1 Shikata-cho, Okayama City, Okayama 700-8558 Japan; 2 Division of Medical Support, Okayama University Hospital, 2-5-1 Shikata-cho, Okayama City, Okayama 700-8558 Japan; 3 Department of Orthopedic Surgery, Okayama Rosai Hospital, 1-10-25 Chikko-Midorimachi, Okayama City, Okayama 702-8055 Japan

**Keywords:** Intraoperative neuromonitoring, Motor evoked potential, Spinal deformity surgery, Alarm point, Propofol, False positive, Anesthetic fade, Abductor digiti minimi, Abductor hallucis, Amplitude

## Abstract

**Background:**

Intraoperative neuromonitoring using motor evoked potentials (MEP) satisfactorily detects motor tract integrity changes during spinal surgery. However, monitoring is affected by “anesthetic fade,” in which the stimulation threshold increases because the waveform amplitude decreases with the accumulation of propofol. Therefore, the purpose of this study was to clarify the effect of anesthetic fade on transcranial MEPs by investigating the time-dependent changes of amplitude during spinal deformity surgeries.

**Methods:**

We retrospectively reviewed medical records of 142 spinal deformity patients (66 patients with idiopathic scoliosis, 28 with adult spinal deformities, 19 with neuromuscular scoliosis, 17 with syndromic scoliosis, and 12 with congenital scoliosis). The average age was 28 years (range, 5 to 81 years). MEPs were recorded bilaterally from the abductor digiti minimi (ADM) and abductor hallucis (AH) muscles during spinal deformity surgeries. The Wilcoxon signed-rank test was used to investigate the time-dependent changes of amplitude after propofol infusion to evaluate anesthetic fade effects.

**Results:**

The average time to baseline from initial propofol infusion was 113 min (range, 45 to 182 min). In the ADM, the amplitude was 52% at 1 h after initial propofol infusion, 102% at 2 h, 105% at 3 h, 101% at 4 h, 86% at 5 h, and 81% at 6 h. Compared to the 2-h time point, MEP decreased significantly by 16% at 5 h (*P* < 0.0005) and by 21% at 6 h (*P* < 0.05). In the AH, the amplitude was 49% at 1 h after initial infusion of propofol, 102% at 2 h, 102% at 3 h, 92% at 4 h, 71% at 5 h, and 63% at 6 h. Compared to the 2-h time point, MEP decreased significantly by 10% at 4 h (*P* < 0.005), by 31% at 5 h (*P* < 0.0000005), and by 39% at 6 h (*P* < 0.05).

**Conclusions:**

MEP amplitude significantly decreased in the upper limbs at 5 and 6 h and in the lower limbs at 4, 5, and 6 h after the initial infusion of propofol, respectively. The influence of anesthetic fade could influence false positive MEPs during long spinal surgeries.

## Background

Iatrogenic spinal cord injury is the most serious complication in scoliosis surgery [1, 2]. The potential for intraoperative neurologic complications has become a particular concern in recent years because patients with many types of deformities are undergoing surgical treatment. As a result, intraoperative neuromonitoring is gaining popularity; therefore, development and thorough understanding of it are necessary to reduce the risk of intraoperative neurologic complications. Transcranial motor evoked potentials (MEP), a type of intraoperative neuromonitoring, has excellent sensitivity for detecting changes in motor tract integrity during spinal surgery [3].

The MEP amplitude can decrease even in the absence of any nerve damage (false positive), and this may be caused by electrode failure, intraoperative hypotension, or hypothermia [4, 5]. In addition, most anesthetics, other than remifentanil and ketamine, can cause false positive results by inhibiting MEP in a dose-dependent fashion [6]. Total intravenous anesthesia using propofol infusion is preferred when MEP monitoring is performed during surgery [7, 8]. However, MEP responses have been observed to deteriorate over the duration of surgery under general anesthesia. MacDonald et al. described “fading MEP” with prolonged exposure to anesthesia [9]. Lyon et al. reported that muscle MEPs tend to exhibit gradually rising thresholds throughout surgery despite stable anesthesia [10].

Spinal deformity surgery usually requires a long operation, so patients are susceptible to anesthetic fade. In addition, corrective forces that have a potential risk of spinal cord damage are usually applied at the final stages of spinal deformity surgery. Therefore, when a decrease in MEP occurs, spine surgeons need to determine whether it is caused by the surgical procedure or anesthetic fade. To our knowledge, the magnitude of amplitude decrease over time due to anesthetic fade has not been quantified in spinal surgery. We retrospectively reviewed a series of patients who underwent spinal deformity surgeries using transcranial MEP monitoring. We measured changes in amplitude over time under stable anesthetic conditions.

## Methods

Following approval from our ethics committee, we reviewed the records of 159 patients who had undergone surgical correction of spinal deformities at our institution from 2013 to 2016. We restricted our study to patients who received maintenance of anesthesia with total intravenous anesthesia during the period of MEP monitoring. We excluded eight cases in which an adequate waveform could not be obtained during the surgery because of preoperative neurological deficits and nine cases in which new neurologic deficits were found after the surgery (i.e., true positive cases) (Table 1). Using these criteria, we included 142 patients who underwent surgical correction for spinal deformities with transcranial MEP monitoring (Table 2).

**Table 1:** Inclusion and exclusion criteria

Inclusion criteria	
Patients who underwent surgical correction for spinal deformities with transcranial MEP monitoring	
Exclusion criteria	
Patients in which an adequate waveform could not be obtained during the surgery	
Patients in which new neurologic deficits were found after the surgery (i.e., true positive cases)

**Table 2:** Types of spinal deformity

Spinal deformity (142 patients)	
Idiopathic scoliosis	66
Adult deformity	28
Neuromuscular scoliosis	19
Syndromic scoliosis	17
Congenital scoliosis	12

The operative reports, anesthesia records, and spinal cord monitoring records were analyzed to determine relationships between duration of anesthesia and consequent loss in amplitude of the MEP and to ascertain the effect of intravenous propofol.

Serial neurophysiological monitoring of spinal cord motor tract function was performed by repeatedly recording both lower and upper extremity efferent transcranial electric motor potentials from the beginning of anesthesia to the end of anesthesia. MEP monitoring was the principal component of a broader neuromonitoring protocol, which included train-of-four (TOF) peripheral nerve stimulation to test for clearance of muscle relaxants from the neuromuscular junction. All neuromonitoring for this study was performed by one experienced neurophysiological monitoring group using a common, algorithmically based standard for both consistency and improved detection accuracy.

### Technique for motor evoked potential monitoring

All patients underwent the standard monitoring techniques for spine surgery at our institution. MEPs were typically recorded from the deltoid, abductor digiti minimi (ADM) of each upper extremity, quadriceps, tibialis anterior, and abductor hallucis (AH) of each lower extremity using surface-patch electrodes (disposable electrode Vitrode V; Nihon Kohden, Japan). In this study, we utilized amplitudes of MEP from the ADM and AH. MEP recordings typically include these muscles, which are known to have strong pyramidal innervation [11].

These myogenic responses were elicited with the use of a commercially available transcranial electrical stimulator (Stimulator SEN-4100; Nihon Kohden, Japan) that delivered an anodal pulse train between two corkscrew electrodes (Coil Electrode SN-100-1500 AD; UNIQUE MEDICAL, Japan) inserted subcutaneously over the motor cortex regions C3-C4 (International 10/20 System). Table 3 shows the MEP measurement parameters.

**Table 3:** Modalities and standardized institutional settings for the assessment of MEP

	MEP stimulation	MEP recording
Monitoring system	Neuromaster MEE-1216 (Nihon Kohden, Japan)	
Electrodes	Corkscrew electrodes (UNIQUE MEDICAL, Japan)	Surface-patch electrodes (Nihon Kohden, Japan)
Montage of electrodes	C3-C4 (according to the international 10/20 system)	Bilateral abductor digiti minimi muscles
		Bilateral abductor hallucis muscles
Specifications	Stimulus (anode), repeated train of 5–7 stimuli	Filters, 0.05–1.5 kHz
	Interstimulus interval, 2 ms	Average, 5
	Intensity, 150–200 mA	
	Duration, 0.5 ms, biphasic	

*MEP* motor evoked potential

### Anesthetic management

**Fig. 1:**
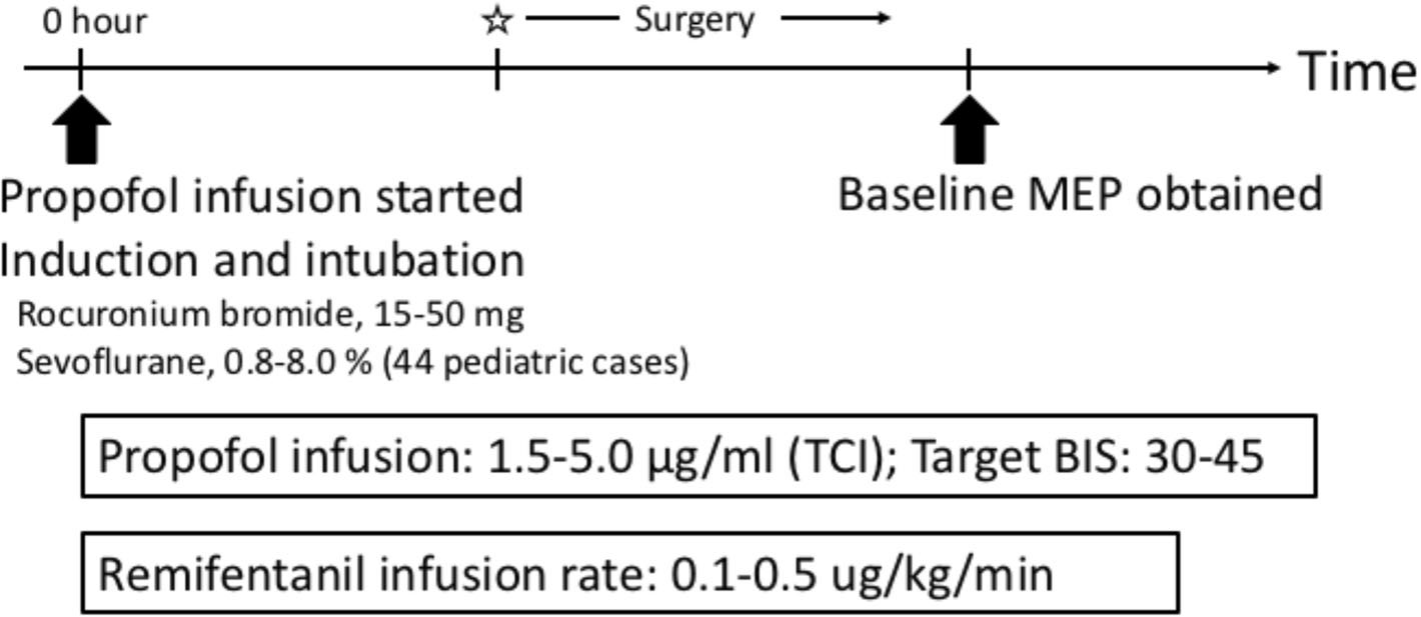
Schema of the time course. Time 0 was defined as when the infusion of propofol started. Muscle relaxant was administered only upon propofol induction. A small amount of inhalation anesthetic (sevoflurane, 0.8 to 8.0%) was used for anesthetic induction in 44 pediatric cases. Anesthesia was maintained with continuous infusion of propofol and remifentanil. Star marker, start of surgery; MEP, motor evoked potential; TCI, target-controlled infusion (a device to deliver the drug to achieve specific predicted target drug concentrations in the blood); BIS, bispectral index (a device to assess the depth of anesthesia)

All patients’ anesthesia was maintained with total intravenous anesthesia during the period of MEP monitoring, as shown in Fig. 1. In 44 pediatric patients, only an initial small amount of inhalation anesthetic (sevoflurane, 0.8% to 8.0%) was used when securing venous access. A muscle relaxant (rocuronium bromide, 15 to 50 mg) was administered before intubation. Following induction and intubation, all inhalational agents were discontinued and no additional muscle relaxants were administered during surgery. An arterial line was placed to monitor blood pressure. Thereafter, general anesthesia was maintained with propofol using target-controlled infusion (TCI; 1.5 to 5 μg/ml) and remifentanil (0.1 to 0.5 μg/kg/min) with particular efforts made to achieve a stable, target range (30 to 45) of the bispectral index (BIS; BIS™ LoC 2 Channel OEM Module; Medtronic, USA) and a mean arterial blood pressure of at least 65 mmHg. The ventilator was set to maintain normocapnia, and the target SpO2 was over 96%. No muscle relaxant was used after intubation so as not to compromise the transcranial electric MEP amplitudes. Monitoring of the neuromuscular junction with TOF stimulation of the posterior tibial nerve and recording compound muscle action potentials from the AH muscle ensured adequate clearance of the neuromuscular blockade. When the TOF was low and the control waveform could not be obtained, reversal of the neuromuscular blockade was performed using sugammadex [12].

### Electrophysiology data analysis

We reviewed the MEP records and investigated the time when the baseline was obtained (the course from the initial infusion of propofol). We set the amplitude of MEP as 100% baseline when the influence of neuromuscular blockade sufficiently disappeared before application of spinal instruments. Recovery from neuromuscular blockade was identified by a TOF > 90% [13]. Then, we reviewed the time-dependent changes of amplitude at 1 h, 2 h, 3 h, 4 h, 5 h, and 6 h after the initial infusion of propofol. About ADM, amplitudes were obtained from 120 cases at 1 h, 127 cases at 2 h, 127 cases at 3 h, 111 cases at 4 h, 75 cases at 5 h, and 18 cases at 6 h. About AH, amplitudes were obtained from 129 cases at 1 h, 139 cases at 2 h, 139 cases at 3 h, 122 cases at 4 h, 86 cases at 5 h, and 21 cases at 6 h. The waveform closest to each time was chosen. Amplitudes of the left and right sides were averaged in ADM and AH, respectively.

We reviewed the time from the initial infusion of propofol to the start of surgery. We investigated the period during which surgery was possible with a stable waveform.

### Statistical analysis

The Wilcoxon signed-rank test was used to compare the changes over time. All *P* values were two-sided, and *P* values of 0.05 or less were considered statistically significant. All statistical analyses were performed with EZR (Kanda, 2012; Saitama Medical Centre, Jichi Medical University, http://www.jichi.ac.jp/saitama-sct/SaitamaHP.files/statmedEN.html), which is a graphical user interface for R (version 2.13.0; The R Foundation for Statistical Computing, Vienna, Austria). More precisely, it is a modified version of R commander (version 1.6–3) that was designed to add statistical functions frequently used in biostatistics.

## Results

**Fig. 2:**
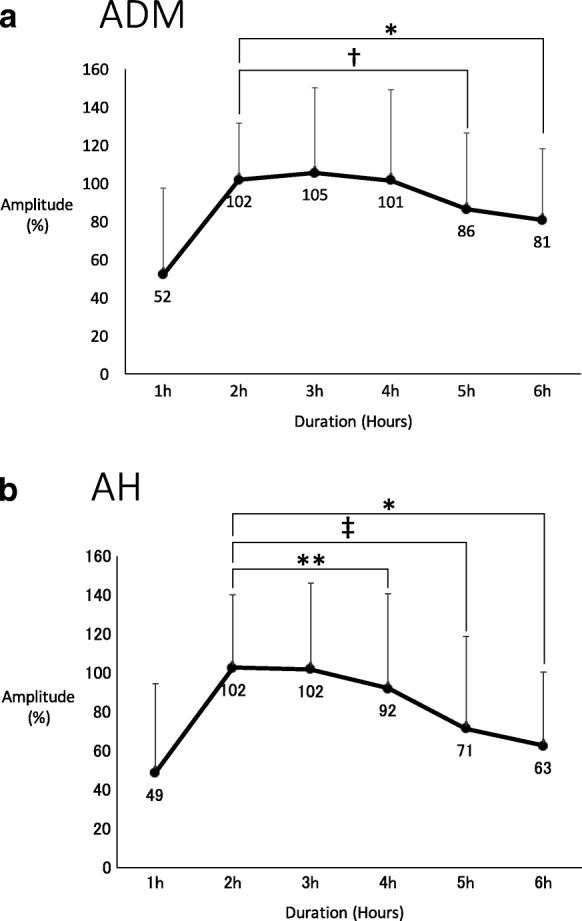
Amplitude changes of the abductor digiti minimi (ADM) and abductor hallucis (AH) muscles post-propofol infusion. **a** In the ADM, the amplitude at 5 and 6 h was significantly lower than that at 2 h. Amplitudes were obtained from 120 cases at 1 h, 127 cases at 2 h, 127 cases at 3 h, 111 cases at 4 h, 75 cases at 5 h, and 18 cases at 6 h. **b** In the AH, the amplitude at 4, 5, and 6 h was significantly lower than at 2 h. Amplitudes were obtained from 129 cases at 1 h, 139 cases at 2 h, 139 cases at 3 h, 122 cases at 4 h, 86 cases at 5 h, and 21 cases at 6 h. (*P < 0.05, **P < 0.005, ^†^*P* < 0.0005, ^‡^*P* < 0.0000005). Values are expressed as means, and bars represent standard deviations

There were 111 (78%) female patients and 31 (22%) male patients ranging in age from 5 to 81 years (average, 28 years) at the time of surgery. The average operating time was 282.1 min (range, 162 to 480 min). One hundred eighteen cases were dorsal operations, 22 cases were ventral operations, and 2 cases were combined ventral and dorsal operations. The average number of levels fused was 9.2 segments (range, 2 to 17 segments). There were no complications related to MEP monitoring. As perioperative comorbidities, surgical site infection (SSI) was identified in 5 cases, postoperative deep vein thrombosis (DVT) in 3 cases, urinary tract infection in 1 case, postoperative delirium in 1 case, and rising hepatic enzyme of unknown cause in 1 case. We performed debridement reoperation for 4 SSI cases. The average time to obtain the baseline from the initial infusion of propofol was 113 min (range, 45 to 182 min). In the upper limbs (ADM), the amplitude was 52% at 1 h after the initial infusion of propofol, 102% at 2 h, 105% at 3 h, 101% at 4 h, 86% at 5 h, and 81% at 6 h. Compared to the 2-h time point (approximation of the time of 113 min we got, the baseline we mentioned earlier), the amplitude at 5 h significantly decreased by 16% (*P* < 0.0005) and that at 6 h significantly decreased by 21% (*P* < 0.05) (Fig. 2a). In the lower limbs (AH), the amplitude was 49% at 1 h after the initial infusion of propofol, 102% at 2 h, 102% at 3 h, 92% at 4 h, 71% at 5 h, and 63% at 6 h. Compared to the 2-h time point, the amplitude at 4 h significantly decreased by 10% (*P* < 0.005), that at 5 h significantly decreased by 31% (*P* < 0.0000005), and that at 6 h significantly decreased by 39% (*P* < 0.05) (Fig. 2b).

The average time from the initial infusion of propofol to the start of surgery was 55 min (range, 0 to 89 min). The waveform decreasing started to occur in 4 h from the start of propofol, and it took about 1 h to start the surgery. Considering those things, MEP varied at 3 h after the start of the surgery.

## Discussion

We reviewed the records of patients who had undergone surgical correction of spinal deformities. We allowed 113 min from the initial infusion of propofol to obtain a sufficient baseline. This seemed to be affected by deep anesthesia during intubation (inhalation anesthetic, propofol, and neuromuscular blockade).

At 4 h after the initial infusion of propofol, the amplitude of MEP decreased by 10% in the lower limbs. At 5 h after the initial infusion of propofol, the amplitude of MEP decreased by 16% in the upper limbs and 31% in the lower limbs. In our cases, the time from the initial infusion of propofol to the start of the surgery averaged 55 min, and then the waveform was stable for only about 2 h (from 1 h to 3 h after the start of surgery). As mentioned previously, spinal deformity surgery usually requires a long operation time. Lonner et al. reported that the operating time for the treatment of adolescent idiopathic scoliosis was 180.2 min for experienced surgeons and 221.9 min for inexperienced surgeons [14]. Taking this into consideration, there is a possibility that a sufficient waveform is not maintained by the end of the surgery. In this study, the average operating time was 282.1 min, because our study includes syndromic scoliosis and advanced deformities that require osteotomy. Such cases may require a longer operation time, and the effect of anesthetic fade may increase.

Anecdotal reports suggest that MEP responses degrade or undergo anesthetic fade during surgery despite unchanged anesthetic levels and other physiologic variables [10]. MEP tends to exhibit gradually rising thresholds during the hours of surgery. Sedative-hypnotics (e.g., propofol) cause unconsciousness by producing corticocortical inhibition, possibly by GABA-mediated inhibitory interneuron activity within the cerebral cortex [15] with minimal depression of spinal alpha motor neurons [16]. Propofol, like halogenated agents, produces a dose-dependent depression of MEP responses [17, 18]. The influence of propofol is also suggested in this study.

In this study, the lower limbs were more affected by anesthetic fade than the upper limbs. This phenomenon has been reported previously [9]. A study about MEP under sevoflurane anesthesia reported that high-frequency stimulation restored MEP to the baseline level in the upper extremities but not in the lower extremities [19]. The authors stated that it remained unclear what mechanism was responsible for the discrepancy in MEP responses between the upper and lower limbs. This discrepancy may have resulted from differences in the corticospinal drive mechanism between the upper and lower limbs; for example, D-wave refractory periods. In MEP under propofol anesthesia, the fade phenomenon may occur with a similar mechanism. Additional basic research is required to answer this question.

With respect to the alarm point of MEP, according to retrospective studies, Pelosi et al. reported a 50% decrease in amplitude [3], and Langeloo et al. reported an 80% decrease in amplitude [20]. According to a prospective multicenter study from the Spinal Cord Monitoring Working Group of the Japanese Society for Spine Surgery and Related Research, they recommend designating a 70% decrease in amplitude as the alarm point for routine spinal cord monitoring, particularly during surgery for spinal deformities, ossification of the posterior longitudinal ligament, and extramedullary spinal cord tumors [21]. The problem with this alarm point is that anesthetic fade is not considered. Other causes of false positives include intraoperative hypothermia and hypotension [4, 5]. In our study, there were no cases of persistent intraoperative hypothermia or hypotension, and the amplitude of the lower limb significantly decreased at 4 h and markedly decreased at 5 h after the initial infusion of propofol. The influence of anesthetic fade could potentially lead to false positive MEPs during long spinal surgeries.

The limitation of this study is that it was a retrospective study, and it included patients with many types of spinal deformities. Spinal cord monitoring in neuromuscular scoliosis is possible but potentially unreliable [22]. The inclusion of patients with neuromuscular scoliosis and patients with mild preoperative paralysis may have influenced our results. We included all cases in which no new postoperative neurological deficits were observed; therefore, we might have included cases with some damage to the spinal cord where no neurological deficits were identified. As another limitation, TCI was used for administration of propofol, and the total amount of propofol administered was unknown. If we know the total amount of propofol administered, it would be possible to take into consideration the amount until the fade phenomenon occurred.

## Conclusions

In summary, the amplitude of MEP significantly decreased in the lower limbs at 4 h and in the upper and lower limbs at 5 h after the initial infusion of propofol. These results may be helpful to reduce the incidence of false positives in MEP monitoring since the influence of anesthetic fade on MEP is inevitable during a long spinal surgery.

## Abbreviations

ADM: Abductor digiti minimi
AH: Abductor hallucis
MEP: Motor evoked potential
TCI: Target-controlled infusion
TOF: Train-of-four
